# Healthcare Provider’s Perceived Self-Efficacy in HPV Vaccination Hesitancy Counseling and HPV Vaccination Acceptance

**DOI:** 10.3390/vaccines11020300

**Published:** 2023-01-30

**Authors:** Ikponmwosa Osaghae, Charles Darkoh, Onyema Greg Chido-Amajuoyi, Wenyaw Chan, Paige Padgett Wermuth, Mala Pande, Sonia A. Cunningham, Sanjay Shete

**Affiliations:** 1 Department of Epidemiology, Human Genetics and Environmental Sciences, The University of Texas Health Science Center at Houston (UTHealth) School of Public Health, Houston, TX 77030, USA; 2 Department of Biostatistics, The University of Texas MD Anderson Cancer Center, Houston, TX 77030, USA; 3 Division of Cancer Prevention and Population Sciences, The University of Texas MD Anderson Cancer Center, Houston, TX 77030, USA; 4 Department of Epidemiology, The University of Texas MD Anderson Cancer Center, Houston, TX 77030, USA; 5 Department of Biostatistics and Data Science, UTHealth School of Public Health, Houston, TX 77030, USA; 6 Department of Management, Policy, and Community Health, UTHealth School of Public Health, Houston, TX 77030, USA; 7 Department of Gastroenterology, Hepatology and Nutrition, The University of Texas MD Anderson Cancer Center, Houston, TX 77030, USA

**Keywords:** human papillomavirus, HPV vaccines, HPV vaccine hesitancy, provider’s self-efficacy, HPV vaccination acceptance

## Abstract

Background: HPV vaccine hesitancy is a key contributor to the sub-optimal HPV vaccination uptake in the United States. We aimed to determine the association between healthcare providers’ self-efficacy in HPV vaccination hesitancy counseling and HPV vaccination acceptance after initial and follow-up counseling sessions. Methods: Population-based cross-sectional study of healthcare providers (HCPs) practicing in Texas. Logistic regression analyses were used to determine the odds of HPV vaccination acceptance by vaccine-hesitant patients. Additionally, generalized estimating equations were used to compare HPV vaccination acceptance by hesitant patients after follow-up versus initial counseling sessions. Results: 1283 HCPs completed the survey with a mean (SD) age of 47.1 (11.3) years. HCPs who believed that they were very/completely confident in counseling HPV-vaccine-hesitant parents had higher odds of observing HPV vaccination acceptance very often/always after an initial counseling session (adjusted odds ratio (AOR): 3.50; 95% CI: 2.25–5.44) and after follow-up counseling sessions (AOR: 2.58; 95% CI: 1.66–4.00) compared to HCPs that perceived they were not at all/somewhat/moderately confident. The odds of HPV vaccination being accepted very often/always by vaccine-hesitant parents was 61% (AOR: 1.61; 95% CI: 1.32–1.95) higher after follow-up counseling sessions compared to an initial counseling session. The results were similar for the counseling of HPV-vaccine-hesitant adult patients. Conclusions: The confidence level of HCPs in counseling hesitant parents and adult patients impacts HPV vaccination acceptance. Importantly, acceptance was higher after follow-up counseling sessions than initial counseling sessions. HCPs should receive training in HPV vaccination counseling to enhance their confidence in counseling hesitant patients and should utilize every visit to counsel hesitant patients.

## 1. Introduction

HPV vaccination is safe and effective as a public health intervention to prevent HPV-associated cancers and minimize HPV-related morbidities and mortalities [1,2,3,4,5]. Since the licensure of the first HPV vaccine for females in 2006 and its subsequent use in males in 2009, over 135 million doses have been administered in the United States (U.S.) [6]. The U.S. Center for Disease Control (CDC) Advisory Committee on Immunization Practices (ACIP) recommends that males and females receive the HPV vaccine from the age of nine up to the age of 26 [7]. It further recommends that adults aged 27 to 45 be vaccinated based on a shared clinical decision between patients and their providers [7]. However, despite the availability of a safe vaccine and clear guidelines on HPV vaccination recommendations by healthcare providers (HCPs), acceptance rates remain suboptimal [8]. In 2020, the average HPV vaccination rate among adolescents aged 13–17 years was just 59% nationally and 55% in Texas [8]. These rates lag far behind the Healthy People 2030 target of 80% and position Texas’ HPV vaccination rate at only 40th among U.S. states [8,9].

The rising HPV vaccine hesitancy among parents of adolescents has been reported as one of the reasons for the low HPV vaccination rate [10,11]. Moreover, the concerns about the safety of HPV vaccines despite overwhelming evidence of HPV vaccine safety and efficacy is a major contributor to HPV vaccine hesitancy by parents and adult patients [6,12,13,14,15]. Similarly, concerns about sexual disinhibition following HPV vaccination are sometimes reported as another reason for HPV vaccine hesitancy by parents and providers despite evidence to the contrary [16,17,18]. Hence, the role of HCP in HPV vaccination counseling is of ever-rising importance. However, despite the effectiveness of HCP recommendations on HPV vaccination, provider recommendations for HPV vaccination remain low [19,20].

In addition to parental or patient factors, provider factors, such as their confidence level and knowledge of HPV vaccines, are notable modifiable factors impacting vaccination coverage rates [21,22,23]. The concept of self-efficacy is an area of extensive research and refers to an individual’s confidence in their ability to execute actions to attain valued goals [24]. An HCP’s self-efficacy in their capability to counsel patients correlates with their delivery of HPV vaccination recommendations and ultimately increases vaccine uptake [22,25]. HCPs tend to recommend HPV vaccination when they feel comfortable counseling their patients about HPV and related concerns [26]. In contrast, providers’ low self-confidence in counseling and discussing safety and sexual concerns relating to HPV vaccines may impede vaccination recommendation and uptake rates [27]. Consequently, understanding the association of HCPs’ self-efficacy in delivering HPV vaccine hesitancy counseling with HPV vaccine uptake could provide valuable insights into designing interventions at the provider level. In addition, it has been established that adopting a healthy behavior involves a process of change that may necessitate exposure to repeated counseling sessions [28]. Moreover, follow-up counseling improves the secondary acceptance of HPV vaccination [29]. Thus, this study aimed to assess the association between provider self-efficacy in their knowledge and ability to counsel HPV-vaccine-hesitant parents of pediatric patients or adult patients and the frequency of HPV vaccination acceptance after initial and follow-up counseling sessions.

## 2. Methods

### Study Design, Data Source, and Population

This was a cross-sectional study from a population-based survey of HCPs in Texas conducted between January and April 2021. The survey was developed and distributed by the University of Texas MD Anderson Cancer Center after a series of testing and feedback from key stakeholders. A pilot test was conducted in December 2020 to ascertain questionnaire usability, comprehensibility, the questions flow, and integrity of skip logic. The target population included all HCPs currently practicing in Texas whose email addresses were available in the LexisNexis Master Provider Referential Database [30]. HCPs were defined as physicians with an MD or equivalent degree in the specialties of internal medicine, family medicine, obstetrics/gynecology, and pediatrics, as well as physician assistants and nurse practitioners. Each invited participant who completed the 10 min online survey was offered a $10 gift card. All participants provided informed consent. The study was approved by the University of Texas MD Anderson Cancer Center Ethical Review Board and followed the Strengthening the Reporting of Observational Studies in Epidemiology (STROBE) guidelines [31].

## 3. Measures

### 3.1. Dependent Variables

#### 3.1.1. HPV Vaccination Acceptance by Vaccine-Hesitant Parents of Pediatric Patients (9–18 Years) after an Initial and Subsequent (Follow-Up) Counseling Sessions

HCPs observed acceptance of HPV vaccination by hesitant parents of pediatric patients (9–18 years) were assessed separately following initial and subsequent (follow-up) counseling sessions based on two survey questions: After you have counseled HPV-vaccine-hesitant parents, please indicate the overall frequency at which they accept vaccination when their child is 9–18 years old: (1) After initial counseling session; and (2) Following subsequent counseling sessions. Possible responses to both questions were “Never”, “Rarely”, “Sometimes”, “Very often”, “Always”, and “Not sure”. Each dependent variable was recategorized as a binary variable, 0 = “Never/Rarely/Sometimes” and 1 = “Very Often/Always”. All “Not sure” responses were dropped.

#### 3.1.2. HPV Vaccination Acceptance by Vaccine-Hesitant Adult Patients (>18 Years) after an Initial and Subsequent (Follow-Up) Counseling Sessions

HCPs observed acceptance of HPV vaccination by vaccine-hesitant adult patients were assessed separately following initial and subsequent (follow-up) counseling sessions, as above, with identical possible responses and recategorization, with the only difference being a modification of the question to consider the older age group: After you have counseled HPV-vaccine-hesitant adult patients (>18 years old), please indicate the overall frequency at which they accept vaccination: (1) After initial counseling session; and (2) Following subsequent counseling sessions.

### 3.2. Independent Variables

#### 3.2.1. HCP’s Perceived Self-Efficacy or Confidence

The primary independent variable was HCP’s perceived self-efficacy or confidence in knowledge and ability to counsel hesitant parents of pediatric patients, as well as hesitant adult patients using the following two questions: (1) How confident are you in your knowledge and ability to counsel parents who are hesitant to vaccinate their child? (2) How confident are you in your knowledge and ability to counsel HPV-vaccine-hesitant adult patients (>18 years)? Possible responses to both questions were “Not at all”, “Somewhat”, “Moderate”, “Very”, or “Completely”. In order to compare HCPs with high self-efficacy to those with low/moderate self-efficacy, we recategorized our independent variables as binary, 0 = “Not at all/Somewhat/Moderate” and 1 = “Very/Completely”.

#### 3.2.2. HCP Socio-Demographic and Practice-Related Factors

We assessed the following HCP-related factors: age (<35 years, 35–54 years, and ≥55 years), sex (male and female), race/ethnicity (non-Hispanic White, non-Hispanic Black, non-Hispanic Other, and Hispanic), and region of practice (urban and rural). We used the zip codes where HCPs primarily worked to determine their region of practice based on the 2013 Rural-Urban Continuum Codes (RUCC) [32]. RUCC codes 1–3 were defined as urban, while 4–9 were defined as rural. Additionally, we assessed the number of years in practice (≤10 years, 11–20 years, and >20 years), the number of patients seen per week (0–50, 51–100, and >100), practice role (physician, nurse, physician assistant, and other), and facility type (solo practice, group practice, university or teaching hospital, Federally Qualified Health Center (FQHC)/public facility, and Other).

## 4. Data Analysis

Descriptive statistics were presented for the independent variables stratified by each dependent variable using proportions and Pearson’s chi-square test for comparison. We had separate logistic regression models for each of our four study outcomes. We predetermined variables for inclusion in our models based on the literature and relevance to our study aim. Multivariable logistic regression analyses were used to estimate the odds of HPV vaccination acceptance by both hesitant parents of pediatric patients, as well as hesitant adult patients after initial and follow-up counseling sessions while adjusting for all covariates included in the final models. Additionally, we examined the association between these covariates and HPV vaccination acceptance by hesitant patients. Furthermore, we estimated the odds of HPV vaccination acceptance by both hesitant parents and adult patients after follow-up counseling sessions compared to the initial counseling session using generalized estimating equations (GEE) with logit link and binomial family. In addition, for our GEE models, we utilized an exchangeable correlation structure with robust standard errors for computation. Multivariable analyses for logistic regression and GEE models were adjusted for HCPs’ age, sex, race/ethnicity, region of practice, years in practice, number of patients seen per week, role in practice, and facility type. All analyses were conducted using Stata/IC Version 15.1. Statistical significance was set as a two-sided *p*-value < 0.05.

## 5. Results

A total of 1283 HCPs completed the survey representing a response rate of 7%. There was no significant difference between respondents and non-respondents with regard to provider type and sex for which data was available for non-respondents. The mean (SD) age of participants was 47.1 (11.3) years. Overall, 966 (77%) of the HCPs were females and 297 (23%) were males. Of the total HCPs, 614 (48%) provided vaccination services to both adult and pediatric patients, 404 (31%) provided vaccination services to only adult patients, and 265 (21%) provided vaccination services to only pediatric patients (See Supplemental Table S1).

### 5.1. Hesitant Parents of Pediatric Patients (9–18 Years): After an Initial Counseling Session

Of the 879 HCPs that provided pediatric services, 548 (62%) responded to the question on the frequency of HPV vaccination acceptance by hesitant parents of pediatric patients after an initial counseling session (Table 1). Of these respondents, 297 (54%) reported that hesitant parents very often/always accepted HPV vaccination, while 251 (46%) observed that hesitant parents never/rarely/sometimes accepted HPV vaccination. HCPs who believed that they were very/completely confident in counseling hesitant parents were more likely to very often/always report HPV vaccination acceptance after an initial counseling session compared to those who were not at all/somewhat/moderately confident (62% vs. 35%; *p*-value: <0.001). Additionally, there was a difference in the distribution of age category (*p*-value: 0.007), practice type (*p*-value: 0.009), and years of practice experience (*p*-value: 0.024) among HCPs that often/always versus never/rarely/sometimes observed HPV vaccination acceptance by hesitant parents after an initial counseling session.

As seen in Table 2, following multivariable logistic regression analyses, HCPs who believed that they were very/completely confident in counseling HPV-vaccine-hesitant parents had three and a half times higher odds (adjusted odds ratio (AOR): 3.50; 95% CI: 2.25–5.44) of observing HPV vaccination acceptance very often/always following an initial counseling session compared to HCPs who believed that they were not at all/somewhat/moderately confident. Additionally, HCPs ≥ 55 years had over two-and-half-fold higher odds (AOR: 2.60; 95% CI: 1.05–6.49) of very often/always observing HPV vaccination acceptance by hesitant parents after an initial counseling session compared to HCPs < 35 years of age.

### 5.2. Hesitant Parents of Pediatric Patients (9–18 Years): After Follow-Up Counseling Sessions

Based on the same 879 HCPs as above, 537 (61%) responded to the question on the frequency of HPV vaccination acceptance by hesitant parents of pediatric patients after follow-up counseling sessions (Table 1). Of these respondents, 348 (65%) reported that hesitant parents very often/always accepted HPV vaccination, while 189 (35%) observed that hesitant parents never/rarely/sometimes accepted HPV vaccination. HCPs who believed that they were very/completely confident in counseling hesitant parents were more likely to very often/always report HPV vaccination acceptance after follow-up compared to those who were not at all/somewhat/moderately confident (71% vs. 49%; *p*-value: <0.001). Additionally, there was a significant difference in the frequency of years of practice experience among HCP that often/always compared to those that never/rarely/sometimes observed HPV vaccination acceptance by hesitant parents after follow-up counseling sessions (*p*-value: 0.049).

Following multivariable logistic regression analyses (Table 2), HCPs who believed that they were very/completely confident in counseling HPV-vaccine-hesitant parents had over two and a half times higher odds (AOR: 2.58; 95% CI: 1.66–4.00) of observing HPV vaccination acceptance very often/always after follow-up counseling sessions compared to HCPs who believed that they were not at all/somewhat/moderately confident. The odds of observing HPV vaccination acceptance very often/always by hesitant parents after follow-up counseling sessions was 94% higher (AOR: 1.94; 95% CI: 1.05–3.58) among Hispanic HCPs compared to non-Hispanic White HCPs. Furthermore, compared to HCPs with ≤10 years, those with 11–20 years of practice experience had 48% lower odds (AOR: 0.52; 95% CI: 0.30–0.89) of observing HPV vaccination acceptance very often/always by hesitant parents after follow-up counseling sessions.

Results of the generalized estimating equation (Table 3) revealed that the odds of HPV vaccination acceptance being very often/always by vaccine-hesitant parents of pediatric patients was 61% higher (AOR: 1.61; 95% CI: 1.32–1.95) after follow-up counseling sessions compared to an initial counseling session.

### 5.3. Hesitant Adult Patients (>18 Years): After an Initial Counseling Session

Of the 1018 HCPs that provided adult services, 462 (45%) responded to the question on the frequency of HPV vaccination acceptance by hesitant adult patients following an initial counseling session (Table 4). Of these respondents, 233 (50%) reported that hesitant adult patients very often/always accepted HPV vaccination, while 229 (50%) observed that hesitant adult patients never/rarely/sometimes accepted HPV vaccination. HCPs who believed that they were very/completely confident in counseling hesitant adult patients were more likely to very often/always report HPV vaccination acceptance following an initial counseling session compared to those who were not at all/somewhat/moderately confident (61% vs. 30%; *p*-value: <0.001).

Following multivariable logistic regression analysis (Table 5), HCPs who believed that they were very/completely confident in counseling HPV-vaccine-hesitant adult patients had over four-fold higher odds (AOR: 4.19; 95% CI: 2.61–6.71) of observing HPV vaccination acceptance very often/always following an initial counseling session compared to HCPs who believed that they were not at all/somewhat/moderately confident. Additionally, non-Hispanic Other HCPs had 82% higher odds (AOR: 1.82; 95% CI: 1.05–3.14) of very often/always observing HPV vaccination acceptance by hesitant adult patients after an initial counseling session compared to Non-Hispanic White HCPs.

### 5.4. Hesitant Adult Patients (>18 Years): After Follow-Up Counseling Sessions

Based on the same 1018 HCPs as above, 456 (45%) responded to the question on the frequency of HPV vaccination acceptance by hesitant adult patients after follow-up counseling sessions (Table 4). Of these respondents, 255 (56%) reported that hesitant adult patients very often/always accepted HPV vaccination compared to 201 (44%) who observed that hesitant adult patients never/rarely/sometimes accepted HPV vaccination. HCPs who believed that they were very/completely confident in counseling hesitant adult patients were more likely to very often/always report HPV vaccination acceptance after follow-up counseling sessions compared to those that were not at all/somewhat/moderately confident (65% vs. 39%; *p*-value: <0.001).

Results of multivariable logistic regression analysis (Table 5) revealed that HCPs who believed that they were very/completely confident in counseling HPV-vaccine-hesitant adult patients had over three-fold higher odds (AOR: 3.05; 95% CI: 1.94–4.79) of observing HPV vaccination acceptance very often/always after follow-up counseling sessions compared to HCPs who believed that they were not at all/somewhat/moderately confident.

Additionally, results of the generalized estimating equation analyses (Table 3) showed that the odds of HPV vaccination acceptance often/always by vaccine-hesitant adult patients was 34% (AOR: 1.34; 95% CI: 1.14–1.57) higher after follow-up counseling sessions compared to an initial counseling session.

## 6. Discussion

In this study, using a sample of HCPs currently practicing in Texas, we found that those perceiving greater self-efficacy in counseling hesitant patients were more likely to observe HPV vaccination acceptance either after an initial counseling session or after follow-up counseling sessions. The latter finding corresponds with a previous study that found a two-time increase in odds of secondary acceptance after a follow-up counseling session from an HCP [29]. These results also align with previous studies indicating a positive association between HCPs’ confidence or knowledge of HPV vaccination and the recommendation and uptake of the HPV vaccine [23,25]. Our study supports current evidence that provider recommendation of HPV vaccination is crucial for uptake [19]. McRee and colleagues found that HCPs confident in addressing parental concerns and questions on the HPV vaccine were more likely to recommend HPV vaccination routinely [25]. In another study by Rutten et al., increased HCP knowledge of the HPV vaccine and vaccination was associated with higher HPV vaccination initiation and completion rates [23]. Together, this body of work provides crucial evidence in support of the potential to increase HPV vaccination acceptance through interventions that focus on improving the self-efficacy of HCPs in counseling hesitant patients. This finding has implications for reducing the burden of HPV-associated cancers and diseases. For example, HPV vaccination has been shown to be associated with a reduced risk of high-grade cervical dysplasia [33].

Notably, the positive association between HCPs’ perceived self-efficacy and observed HPV vaccination acceptance after initial and follow-up counseling sessions held true for hesitant parents of pediatric patients, as well as for hesitant adult patients. Indeed, since HPV vaccination acceptance is higher among adolescents than young adults, our finding points to the far-reaching effect of providers’ self-efficacy in increasing HPV vaccination acceptance across different patient demography [34,35]. We also assessed the influence of various provider-level characteristics, including provider age, race/ethnicity, experience, and practice type on HPV vaccination acceptance given that these variables are known predictors of HPV vaccination recommendation, a key determinant of vaccination acceptance [36]. Additionally, provider experience acquired either from years of counseling hesitant patients or from seeing a large number of patients is an essential factor associated with the self-efficacy of HCPs in counseling HPV-vaccine-hesitant patients [37]. However, while our study showed a consistent association between provider’s self-efficacy and the acceptance of the HPV vaccination across all sub-groups assessed, we did not see a similar association between HCP’s age, years of experience, or practice type and the observed acceptance of HPV vaccination after initial and follow-up counseling sessions for parents of pediatric patients and adult patients. Specifically, our study showed that older HCPs were more likely to observe HPV vaccination acceptance by hesitant parents after an initial counseling session. This may be due to an increased level of confidence in counseling HPV-vaccine-hesitant patients that comes with experience from years of practice and counseling many patients [38]. This is consistent with the well-established self-efficacy theory, which suggests that individuals who feel confident in their ability to perform a given action successfully will likely continue to engage in that activity (counseling vaccine-hesitant parents and adult patients) [24]. Additionally, Hispanic HCPs were more likely to observe HPV vaccination acceptance by hesitant parents after follow-up counseling sessions. This may be related to the higher HPV vaccination rates among Hispanics [8].

Our study also revealed that hesitant parents of pediatric patients and adult patients are more likely to accept HPV vaccination following repeated counseling sessions than an initial counseling session. This finding supports a previous study, which revealed that patients’ interaction with the healthcare system and regular visits to a provider are associated with HPV vaccination initiation [39]. In addition, this finding aligns with the transtheoretical model of change, which suggests that change is neither instantaneous nor linear but rather that individuals go through processes of change before adopting a healthy behavior [28]. Furthermore, our study counters and provides a valuable alternative to the controversial dismissal policy (letting go or removing hesitant patients from a practice) as a strategy for curbing vaccine hesitancy [40,41]. Nationally, over a fifth of pediatricians dismiss hesitant patients who refuse at least one vaccine [41]. Additionally, a quarter of north Texas providers support dismissal policies after repeated counseling attempts [42]. Although an evaluation of the number of counseling sessions predictive of HPV vaccine uptake is beyond the scope of this study, HPV-vaccine-hesitant patients or their caregivers may benefit from repeated counseling sessions to ease their concerns, overcome long-held beliefs, and allow progress through the various stages of change before they potentially accept the HPV vaccination.

Furthermore, our study has important implications for increasing HPV vaccination by targeting provider-level factors such as self-efficacy in counseling hesitant patients. HCPs would benefit from HPV vaccination training in line with the CDC ACIP guidelines for providers to counsel and recommend the HPV vaccines to their patients at every clinical encounter regardless of whether they were counseled but refused vaccination during their previous clinic visits [7]. Various training approaches have been found to be effective in increasing provider self-efficacy in counseling patients, improving motivation to recommend HPV vaccination, and increasing HPV vaccination initiation among adolescents [36,43,44,45,46]. For example, the use of a training module is effective in increasing knowledge and self-efficacy in HPV-related disease and vaccination counseling [44]. Additionally, continued online education programs for HCPs have increased their self-efficacy in addressing common HPV-related parental concerns about safety, fertility, and the young vaccination age [47]. Given the increasing burden of HPV-associated cancers, there is a need to incorporate HPV vaccination counseling training for HCPs into the current curriculums and continuous medical education for HCPs.

Our study has some limitations. This was a cross-sectional study; as such, we are unable to infer causality. In addition, HPV vaccination acceptance was the perception of HCPs and may not reflect actual event rates. This study was designed from the perspective of HCPs who reported changes in HPV vaccination acceptance among pediatric and adult patients. Given that we did not directly sample patients, our study cannot provide insights into HCP’s self-efficacy in counseling hesitant pediatric patients transitioning into adulthood (≥18 years) between initial and follow-up counseling sessions. Additionally, there is the potential for recall bias as HCPs had to recollect their experiences in the past. Furthermore, this study may be prone to residual confounding from unmeasured covariates. For example, we did not account for time-changing variables, such as HCP training, that may have occurred between initial and follow-up counseling sessions. However, the generalizability of our results is increased by using a statewide survey of HCPs in Texas.

## 7. Conclusions

In conclusion, we found that HCPs more confident in counseling hesitant parents and adult patients were more likely to observe HPV vaccination acceptance after an initial or follow-up counseling session. However, HPV vaccination acceptance was more frequent after follow-up counseling sessions compared to an initial counseling session. HCPs’ age and race/ethnicity were associated with HPV vaccination acceptance when counseling hesitant parents. HCPs should utilize every opportunity to counsel HPV-vaccine-hesitant patients. Additionally, HCPs will benefit from interventions to enhance their self-efficacy in counseling hesitant patients and their caregivers. To better understand the effect of multiple counseling sessions on HPV vaccination acceptance, future studies should evaluate the number of counseling sessions predictive of HPV vaccination acceptance by hesitant patients.

## Figures and Tables

**Table 1:** Distribution of self-efficacy of HCPs, HCP-demographic and practice-related factors for HCPs by strata of frequency of HPV vaccination acceptance by hesitant parents of pediatric patients (9–18 years) after initial and follow-up counseling sessions.

	HPV Vaccination Acceptance by Hesitant Parents of Pediatric Patients (9–18 Years)					
	Initial Counseling Session (*n* = 548)			Follow-Up Counseling Session (*n* = 537)		
HCP Characteristics	Never/ Rarely/ Sometimes (*n* = 251)	Very Often/ Always (*n* = 297)	*p*-Value	Never/ Rarely/ Sometimes (*n* = 189)	Very Often/ Always (*n* = 348)	*p*-Value
**Self-efficacy, *n* (%)**			<0.001			<0.001
Not at all/Somewhat/Moderate	98 (64.9)	53 (35.1)		75 (51.0)	72 (49.0)	
Very/Completely	152 (38.5)	243 (61.5)		114 (29.4)	274 (70.6)	
**Provider age, years, *n* (%)**			0.007			0.555
<35	34 (58.6)	24 (41.4)		23 (41.1)	33 (58.9)	
35–54	164 (47.7)	180 (52.3)		120 (35.3)	220 (64.7)	
≥55	51 (35.9)	91 (64.1)		45 (32.9)	92 (67.2)	
**Sex, *n* (%)**			0.897			0.207
Female	189 (46.1)	221 (53.9)		136 (33.9)	265 (66.1)	
Male	60 (45.5)	72 (54.6)		52 (40.0)	78 (60.0)	
**Race/Ethnicity, *n* (%)**			0.410			0.120
Non-Hispanic White	126 (46.3)	146 (53.7)		100 (37.3)	168 (62.7)	
Non-Hispanic Black	23 (52.3)	21 (47.7)		16 (36.4)	28 (63.6)	
Hispanic	33 (37.9)	54 (62.1)		19 (23.2)	63 (76.8)	
Non-Hispanic Other	59 (45.4)	71 (54.6)		47 (36.4)	82 (63.6)	
**Practice location, *n* (%)**			0.186			0.436
Rural	9 (33.3)	18 (66.7)		7 (28.0)	18 (72.0)	
Urban	241 (46.4)	279 (53.7)		182 (35.6)	329 (64.4)	
**Provider type, *n* (%)**			0.397			0.283
Physician	146 (43.6)	189 (56.4)		109 (32.7)	224 (67.3)	
Nurse	64 (47.8)	70 (52.2)		51 (40.2)	76 (59.8)	
Physician Assistant	31 (49.2)	32 (50.8)		21 (34.4)	40 (65.6)	
Other	10 (62.5)	6 (37.5)		8 (50.0)	8 (50.0)	
**Type of practice, *n* (%)**			0.009			0.244
University/Teaching hospital	55 (47.0)	62 (53.0)		43 (37.4)	72 (62.6)	
Solo practice	24 (39.3)	37 (60.7)		21 (35.0)	39 (65.0)	
Group practice	97 (42.5)	131 (57.5)		74 (33.0)	150 (67.0)	
FQHC/Public facility	32 (41.6)	45 (58.4)		21 (28.8)	52 (71.2)	
Other	43 (66.2)	22 (33.9)		30 (46.2)	35 (53.8)	
**Years in practice, *n* (%)**			0.024			0.049
≤10 years	92 (50.0)	92 (50.0)		60 (33.2)	121 (66.9)	
11–20 years	96 (49.7)	97 (50.3)		79 (42.0)	109 (58.0)	
>20 years	63 (37.3)	106 (62.7)		50 (30.1)	116 (69.9)	
**No of patients seen (per week), *n* (%)**			0.432			0.171
≤50	81 (46.8)	92 (53.2)		66 (39.1)	103 (61.0)	
51–100	129 (47.1)	145 (52.9)		95 (35.2)	175 (64.8)	
>100	36 (39.6)	55 (60.4)		24 (27.3)	64 (72.7)	

Missing observations from initial counseling sessions: self-efficacy, 2; age, 4; sex, 6; race/ethnicity, 15; practice location, 1; years in practice, 2; number of patients seen, 10. Missing observations from follow-up counseling sessions: as above except for race/ethnicity, 14.

**Table 2:** Logistic regression analyses of the association between HCPs’ self-efficacy, socio-demographic, and practice-related factors with acceptance of HPV vaccination by hesitant parents of pediatric patients (9–18 years) after initial and follow-up counseling sessions.

	HPV Vaccination Acceptance by Hesitant Parents of Pediatric Patients (9–18 Years)			
	Initial Counseling Session		Follow-Up Counseling Sessions	
HCP Characteristics	Adjusted OR (95% CI)	*p*-Value	Adjusted OR (95% CI)	*p*-Value
**Self-efficacy**				
Not at all/Somewhat/Moderate	Ref.	Ref.	Ref.	Ref.
Very/Completely	3.50 (2.25–5.44)	<0.001	2.58 (1.66–4.00)	<0.001
**Provider age, years**				
<35	Ref.	Ref.	Ref.	Ref.
35–54	1.49 (0.74–3.00)	0.264	1.80 (0.88–3.71)	0.109
≥55	2.60 (1.05–6.49)	0.040	2.01 (0.78–5.18)	0.150
**Sex**				
Female	Ref.	Ref.	Ref.	Ref.
Male	1.06 (0.67–1.67)	0.816	0.71 (0.45–1.14)	0.160
**Race/Ethnicity**				
Non-Hispanic White	Ref.	Ref.	Ref.	Ref.
Non-Hispanic Black	0.91 (0.44–1.88)	0.796	0.82 (0.39–1.71)	0.591
Hispanic	1.50 (0.87–2.59)	0.146	1.94 (1.05–3.58)	0.033
Non-Hispanic Other	1.22 (0.76–1.96)	0.401	1.12 (0.69–1.81)	0.642
**Practice location**				
Rural	Ref.	Ref.	Ref.	Ref.
Urban	0.69 (0.27–1.74)	0.432	0.76 (0.28–2.06)	0.585
**Provider type**				
Physician	Ref.	Ref.	Ref.	Ref.
Nurse	0.95 (0.59–1.53)	0.837	0.71 (0.43–1.16)	0.172
Physician Assistant	0.93 (0.50–1.75)	0.832	0.96 (0.50–1.83)	0.891
Other	0.37 (0.12–1.12)	0.078	0.40 (0.14–1.19)	0.099
**Type of practice**				
University/Teaching hospital	Ref.	Ref.	Ref.	Ref.
Solo practice	1.09 (0.53–2.23)	0.818	0.78 (0.38–1.63)	0.510
Group practice	1.10 (0.64–1.88)	0.740	1.02 (0.59–1.78)	0.940
FQHC/Public facility	1.11 (0.57–2.16)	0.765	1.27 (0.63–2.59)	0.506
Other	0.42 (0.21–0.86)	0.018	0.58 (0.28–1.17)	0.125
**Years in practice**				
≤10 years	Ref.	Ref.	Ref.	Ref.
11–20 years	0.76 (0.46–1.26)	0.280	0.52 (0.30–0.89)	0.017
>20 years	0.78 (0.40–1.53)	0.471	0.77 (0.38–1.59)	0.482
**No of patients seen (per week)**				
≤50	Ref.	Ref.	Ref.	Ref.
51–100	0.73 (0.47–1.15)	0.174	0.96 (0.61–1.52)	0.875
>100	0.93 (0.49–1.74)	0.812	1.50 (0.77–2.93)	0.235

OR: odds ratio; CI: confidence interval; Ref: reference.

**Table 3:** HPV vaccination acceptance by hesitant parents of pediatric patients (9–18 years) and adult patients (>18 years) after follow-up compared to an initial counseling session. Results of generalized estimating equations.

	HPV Vaccination Acceptance by Hesitant Parents of Pediatric Patients (9–18 Years)				HPV Vaccination Acceptance by Hesitant Adult Patients (>18 Years)			
Characteristics	Crude OR (95% CI)	*p*-Value	^a^ Adjusted OR (95% CI)	*p*-Value	Crude OR (95% CI)	*p*-Value	^a^ Adjusted OR (95% CI)	*p*-Value
**Counseling sessions**								
Initial	Ref.	Ref.	Ref.	Ref.	Ref.	Ref.	Ref.	Ref.
Follow-up	1.55 (1.31–1.84)	<0.001	1.61 (1.32–1.95)	<0.001	1.27 (1.11–1.46)	0.001	1.34 (1.14–1.57)	<0.001

OR: odds ratio; CI: confidence interval; Ref: reference. ^a^ Generalized estimating equation models adjusted for HCP’s self-efficacy, age, sex, race/ethnicity, location of practice, provider type, practice type, number of years in practice, and number of patients seen.

**Table 4:** Distribution of self-efficacy of HCPs, HCP-demographic and practice-related factors for HCPs by strata of frequency of HPV vaccination acceptance by hesitant adult patients (>18 years) after initial and follow-up counseling sessions.

	HPV Vaccination Acceptance by Hesitant Adult Patients (>18 Years)					
	Initial Counseling Session (*n* = 462)			Follow-Up Counseling Session (*n* = 456)		
HCP Characteristics	Never/ Rarely/ Sometimes (*n* = 229)	Very Often/ Always (*n* = 233)	*p*-Value	Never/ Rarely/ Sometimes (*n* = 201)	Very Often/ Always (*n* = 255)	*p*-Value
**Self-efficacy, *n* (%)**			<0.001			<0.001
Not at all/Somewhat/Moderate	110 (70.5)	46 (29.5)		94 (61.4)	59 (38.6)	
Very/Completely	118 (38.8)	186 (61.2)		106 (35.2)	195 (64.8)	
**Provider age, years, *n* (%)**			0.421			0.094
<35	32 (58.2)	23 (41.8)		32 (57.1)	24 (42.9)	
35–54	137 (48.9)	143 (51.1)		119 (43.4)	155 (56.6)	
≥55	59 (48.4)	63 (51.6)		48 (40.0)	72 (60.0)	
**Sex, *n* (%)**			0.086			0.151
Female	163 (47.4)	181 (52.6)		143 (42.2)	196 (57.8)	
Male	63 (56.8)	48 (43.2)		55 (50.0)	55 (50.0)	
**Race/Ethnicity, *n* (%)**			0.182			0.342
Non-Hispanic White	125 (52.3)	114 (47.7)		109 (45.8)	129 (54.2)	
Non-Hispanic Black	22 (59.5)	15 (40.5)		19 (51.4)	18 (48.7)	
Hispanic	30 (42.3)	41 (57.8)		23 (35.4)	42 (64.6)	
Non-Hispanic Other	46 (44.2)	58 (55.8)		44 (41.5)	62 (58.5)	
**Practice location, *n* (%)**			0.180			0.993
Rural	10 (37.0)	17 (63.0)		11 (44.0)	14 (56.0)	
Urban	219 (50.3)	216 (49.7)		190 (44.1)	241 (55.9)	
**Provider type, *n* (%)**			0.813			0.773
Physician	107 (47.4)	119 (52.7)		99 (44.4)	124 (55.6)	
Nurse	76 (51.0)	73 (49.0)		63 (42.6)	85 (57.4)	
Physician Assistant	37 (52.9)	33 (47.1)		30 (43.5)	39 (56.5)	
Other	9 (52.9)	8 (47.1)		9 (56.3)	7 (43.8)	
**Type of practice, *n* (%)**			0.203			0.095
University/Teaching hospital	53 (45.3)	64 (54.7)		46 (39.3)	71 (60.7)	
Solo practice	25 (61.0)	16 (39.0)		20 (48.8)	21 (51.2)	
Group practice	87 (51.8)	81 (48.2)		82 (48.8)	86 (51.2)	
FQHC/Public facility	29 (40.9)	42 (59.2)		21 (31.8)	45 (68.2)	
Other	35 (53.9)	30 (46.2)		32 (50.0)	32 (50.0)	
**Years in practice, *n* (%)**			0.115			0.122
≤10 years	92 (54.4)	77 (45.6)		81 (48.8)	85 (51.2)	
11–20 years	79 (50.6)	77 (49.4)		70 (45.5)	84 (54.6)	
>20 years	57 (42.5)	77 (57.5)		49 (37.1)	83 (62.9)	
**No of patients seen (per week), *n* (%)**			0.119			0.543
≤50	82 (48.2)	88 (51.8)		74 (44.6)	92 (55.4)	
51–100	111 (47.8)	121 (52.2)		98 (42.6)	132 (57.4)	
>100	34 (63.0)	20 (37.0)		27 (50.9)	26 (49.1)	

Missing observations from initial counseling sessions: self-efficacy, 2; age, 5; sex, 7; race/ethnicity, 11; years in practice, 3; number of patients seen, 6. Missing observations from follow-up counseling sessions: self-efficacy, 2; age, 6; sex, 7; race/ethnicity, 10; years in practice, 4; number of patients seen, 7.

**Table 5:** Logistic regression analyses of the association between HCPs’ self-efficacy, socio-demographic and practice-related factors with acceptance of HPV vaccination by hesitant adult patients (>18 years) after initial and follow-up counseling sessions.

	HPV Vaccination Acceptance by Hesitant Adult Patients (>18 Years)			
	Initial Counseling Session		Follow-Up Counseling Sessions	
HCP Characteristics	Adjusted OR (95%CI)	*p*-Value	Adjusted OR (95% CI)	*p*-Value
**Self-efficacy**				
Not at all/Somewhat/Moderate	Ref.	Ref.	Ref.	Ref.
Very/Completely	4.19 (2.61–6.71)	<0.001	3.05 (1.94–4.79)	<0.001
**Provider age, years**				
<35	Ref.	Ref.	Ref.	Ref.
35–54	1.00 (0.49–2.06)	0.999	1.43 (0.71–2.88)	0.323
≥55	0.90 (0.34–2.39)	0.826	1.52 (0.58–3.96)	0.393
**Sex**				
Female	Ref.	Ref.	Ref.	Ref.
Male	0.65 (0.39–1.08)	0.095	0.76 (0.46–1.26)	0.292
**Race/Ethnicity**				
Non-Hispanic White	Ref.	Ref.	Ref.	Ref.
Non-Hispanic Black	0.79 (0.35–1.78)	0.564	0.80 (0.36–1.77)	0.589
Hispanic	1.63 (0.90–2.97)	0.108	1.49 (0.80–2.75)	0.208
Non-Hispanic Other	1.82 (1.05–3.14)	0.031	1.45 (0.85–2.45)	0.169
**Practice location**				
Rural	Ref.	Ref.	Ref.	Ref.
Urban	0.60 (0.23–1.52)	0.279	0.92 (0.36–2.35)	0.869
**Provider type**				
Physician	Ref.	Ref.	Ref.	Ref.
Nurse	0.74 (0.45–1.22)	0.241	1.11 (0.68–1.81)	0.685
Physician Assistant	0.83 (0.44–1.56)	0.553	1.22 (0.65–2.28)	0.529
Other	0.90 (0.30–2.67)	0.849	0.62 (0.21–1.87)	0.400
**Type of practice**				
University/Teaching hospital	Ref.	Ref.	Ref.	Ref.
Solo practice	0.62 (0.27–1.41)	0.254	0.69 (0.31–1.52)	0.351
Group practice	0.91 (0.51–1.60)	0.738	0.70 (0.40–1.22)	0.208
FQHC/Public facility	1.27 (0.64–2.53)	0.500	1.32 (0.64–2.71)	0.447
Other	0.81 (0.41–1.60)	0.539	0.64 (0.32–1.27)	0.201
**Years in practice**				
≤10 years	Ref.	Ref.	Ref.	Ref.
11–20 years	1.21 (0.70–2.10)	0.499	0.97 (0.56–1.68)	0.919
>20 years	1.51 (0.70–3.22)	0.292	1.27 (0.60–2.70)	0.536
**No. of patients seen (per week)**				
≤50	Ref.	Ref.	Ref.	Ref.
51–100	0.78 (0.49–1.25)	0.309	0.85 (0.53–1.35)	0.493
>100	0.52 (0.24–1.13)	0.098	0.86 (0.41–1.80)	0.686

OR: odds ratio; CI: confidence interval; Ref.: reference.

## Data Availability

The data presented in this study are available on request from the corresponding author.

## Supplementary Materials

The following supporting information can be downloaded at: https://www.mdpi.com/article/10.3390/vaccines11020300/s1, Table S1: Descriptive statistics of the overall population of HCPs (N = 1283).
