# Supplementary material for: Healthcare Provider’s Perceived Self-Efficacy in HPV Vaccination Hesitancy Counseling and HPV Vaccination Acceptance
Source: Vaccines (Basel). 2023 Jan 30;11(2):300. doi: 10.3390/vaccines11020300 (PMC9965421; doi:10.3390/vaccines11020300)
Supplement: Supplementary file 1 [file vaccines-11-00300-s001.zip › vaccines-2133129-supplementary.pdf]

Supplementary Table S1. Descriptive statistics of the overall population of HCPs (N = 1283).

HCP Characteristics	n (%)
**Provider age, years[a]**	
< 35	160 (12.7)
35-54	783 (62.2)
≥ 55	316 (25.1)
**Sex**	
Female	966 (76.5)
Male	297 (23.5)
**Race/Ethnicity**	
Non-Hispanic White	668 (53.3)
Non-Hispanic Black	116 (9.3)
Hispanic	164 (13.1)
Non-Hispanic Other	306 (24.4)
**Practice location**	
Rural	54 (4.2)
Urban	1228 (95.8)
**Provider type**	
Physician	501 (39.1)
Nurse	407 (31.7)
Physician Assistant	273 (21.3)
Other	102 (8.0)
**Type of practice**	
University/Teaching hospital	398 (31.0)
Solo practice	144 (11.2)
Group practice	407 (31.7)
FQHC/Public facility	132 (10.3)
Other	202 (15.7)
**Years in practice**	
≤ 10 years	492 (38.7)
11-20 years	434 (34.2)
> 20 years	344 (27.1)
**No of patients seen (per week)**	
≤ 50	608 (48.8)
51-100	486 (39.0)
> 100	153 (12.3)
**Type of patients seen**	
Both adults and children	614 (47.9)
Only Adults	404 (31.4)
Only children	265 (20.7)

[a]Mean (SD) provider age:47.1 (11.3).

SD = Standard deviation.

Missing observations: age, 24; sex, 20; practice location, 1; race/ethnicity, 29; years in practice, 13; number of patients seen per week, 36.
